# Genetic diversity and characterization of arsenic-resistant endophytic bacteria isolated from *Pteris vittata*, an arsenic hyperaccumulator

**DOI:** 10.1186/s12866-018-1184-x

**Published:** 2018-05-08

**Authors:** Yunfu Gu, Yingyan Wang, Yihao Sun, Ke Zhao, Quanju Xiang, Xiumei Yu, Xiaoping Zhang, Qiang Chen

**Affiliations:** Department of Microbiology, College of Resource Science and Technology, Sichuan Agricultural University, Chengdu, 611130 China

**Keywords:** *Pteris vittata*, Endophytic bacteria, Genetic diversity, Arsenite resistance, Arsenic transport genes, Horizontal gene transfer

## Abstract

**Background:**

Alleviating arsenic (As) contamination is a high-priority environmental issue. Hyperaccumulator plants may harbor endophytic bacteria able to detoxify As. Therefore, we investigated the distribution, diversity, As (III) resistance levels, and resistance-related functional genes of arsenite-resistant bacterial endophytes in *Pteris vittata* L. growing in a lead-zinc mining area with different As contamination levels.

**Results:**

A total of 116 arsenite-resistant bacteria were isolated from roots of *P. vittata* with different As concentrations. Based on the 16S rRNA gene sequence analysis of representative isolates, the isolates belonged to *Proteobacteria*, *Actinobacteria*, and *Firmicutes*. Major genera found were *Agrobacterium*, *Stenotrophomonas*, *Pseudomonas*, *Rhodococcus*, and *Bacillus*. The most highly arsenite-resistant bacteria (minimum inhibitory concentration > 45 mM) were isolated from *P. vittata* with high As concentrations and belonged to the genera *Agrobacterium* and *Bacillus*. The strains with high As tolerance also showed high levels of indole-3-acetic acid (IAA) production and carried *arsB*/*ACR3(2)* genes. The *arsB* and *ACR3*(*2*) were most likely horizontally transferred among the strains.

**Conclusion:**

The results of this study suggest that *P. vittata* plants with high As concentrations may select diverse arsenite-resistant bacteria; this diversity might, at least partly, be a result of horizontal gene transfer. These diverse endophytic bacteria are potential candidates to enhance phytoremediation techniques.

## Background

Arsenic (As) is a trace metalloid element present in various soil and water ecosystems and originated from both natural processes and human activities [1]. Chronic exposure to soil As possess potential health risks to environment and human health because of its toxicity and carcinogenicity [2]. Moreover, soil contamination with As affects the physiology, growth, and grain quality of crops. For example, the As concentrations in rice grains from Chenzhou, China, exceeded the maximum allowable value of 0.5 mg kg^− 1^ (dry weight) [3]. However, the efficient remediation of As-contaminated soil and water is a major environmental challenge, calling for the development of sophisticated remediation strategies.

Phytoremediation employs plants to accumulate, transfer, stabilize, and remove trace elements from polluted soils and water systems. It has received considerable attention due to its economic and environmental benefits [4]. However, there are several limiting factors affecting the effectiveness of phytoremediation, including plant growth rate, contaminant phytotoxicity, root biomass decrease, and limited uptake of contaminants [5]. In terms of suitable plant species, the arsenic hyperaccumulator plant *P. vittata.* L (Chinese brake fern) has potential to be used in the phytoremediation of As-contaminated soils, since it is able to accumulate more than 1000 mg kg^− 1^ of As in its fronds [6]. Plant-associated microbes could affect the efficiency of metal extraction by plants and thereby enhance phytoremediation processes [7, 8]. They play an important role in the transformation of arsenic, including arsenite (As(III)) oxidation, arsenate (As(V)) respiration, and As(V) reduction, thus affecting the bioavailability and toxicity of As in soils [9]. However, our knowledge about endophytic microorganisms associated with *P. vittata* and their roles in As tolerance and transformation is severely limited.

Endophytic bacteria, which have great potential in enhancing phytoremediation processes [7, 10], can colonize internal plant parts, without any detrimental effects on the hosts [11]. Such bacteria are diverse and influenced by edaphic properties and contaminant contents of both the soil and plant tissue [12, 13]. Some endophytes can promote plant growth by improving nutrient uptake and increasing resistance to metals, suggesting their application in phytoremediation [14]. Bacteria have developed different strategies to transform arsenic, including cytoplasmic arsenate reduction, arsenite oxidation, respiratory arsenate reduction, and arsenite methylation [15]. The reduction of arsenate, followed by the extrusion (efflux) of arsenite, is the main mechanism of arsenate tolerance in bacteria [16]. Arsenite efflux is carried out by membrane carrier proteins or pumps, such as ArsB or ArsAB complexes and Acr3p. Although the arsenite transporter genes *arsB* and *Acr3p* have been identified in various soil bacterial species [17], knowledge about these genes in endophytic bacteria is still lacking. Moreover, the relation between genotypes and arsenite resistance levels has not been addressed. Thus, studying the diversity and distribution of indigenous bacterial endophytes in *P. vittata* is crucial to improve phytoremediation strategies for As-contaminated sites.

In this context, we isolated endophytic bacteria from *P. vittata* from a Pb-Zn mine soil for analysis of the 16S rRNA gene and arsenite transporter gene (e.g. *arsB* and *Acr3p*) diversity. The objectives of this study were to (1) evaluate the distribution and diversity of arsenite-resistant endophytic bacteria in *P. vittata* with different arsenic contamination levels; and (2) investigate the arsenite transporter genes and correlate their presence to the arsenic resistance level of the bacteria. We hypothesized that the distribution and diversity of endophytic bacteria and their arsenite transporter genes are related to the As concentration in *P. vittata* roots.

## Methods

### Ethics statement

No specific permits were required for the described studies. No specific permissions were required for these locations/activities because sample collection did not involve endangered or protected species or privately owned location.

### Study sites and sampling strategy

Sampling was performed in the Tangjia Pb-Zn mine in Hanyuan, Sichuan (29°24′39“ N, 102° 39′ 24” E), 890 m above sea level. The area has a mean annual temperature of 18.5 °C, with a mean annual precipitation of 486 mm and an average annual evaporation of 1553 mm. Based on the findings of a previous field survey, *P. vittata* is the dominant species in this area [18].

With regard to the distribution of the slag heaps, four different soils within the mining area, with different arsenic contamination levels, were selected: an ore outlet (site S2) and a tailing dam with smelting wastes and wastewater (site S3) with high As-contamination levels, and an ore charge heap (site S4) and a slag heap (site S5) with intermediate levels. A field used to cultivate summer rice and winter wheat, approximately 10 km from the mine area, was chosen as a non-contaminated control site (site S1). Soil and ferns samples (reproductive stage) were collected in August 2016 with the permission of the land owner. For this, three plots of 16 m × 10 m were randomly established in each site; each plot was divided into four 8 m × 5 m sampling subplots. Fresh roots and the corresponding rhizosphere soils from three individual *P. vittata* plants were sampled from each subplot. The samples from the subplots were pooled and homogenized to form a composite plot-level sample.

Root and soil samples were placed in polyethylene bags at 4 °C and transported to the laboratory for analyses. For metal analysis, roots were washed with distilled water and dried to constant weight at 55 °C. Dried roots were ground into a fine powder and digested with HNO_3_/HClO_4_ (87/13 *v*/v). Soil samples were air-dried at ambient room temperature (25 °C) and sieved through a 6-mm sieve. Then metals in the soil samples were extracted with *aqua regia*. Total concentrations of As, Pb, Zn, Cu, and Cd in root extractant and *aqua regia* were determined using an inductively coupled-plasma optical emission spectrometer (ICP-AES, IRIS Intrepid II, Thermo Electron, USA) [19]. Soil pH was determined with a potentiometry method, using a soil-to-water ratio of 1:5 [20]. Soil water content (WC) was determined after oven-drying at 105 °C for 48 h [20]. Soil total nitrogen (TN) and organic carbon (SOC) were analyzed via the potassium dichromate oxidation-external heating method and the alkaline hydrolysis diffusion method, respectively [20].

### Isolation of as-resistant endophytic bacterial strains

The *P. vittata* root samples were washed with tap water, followed by three rinses with deionized water and sterilization by sequential immersion in 70% (*v*/v) ethanol for 2 min and 1% mercuric chloride for 1 min; subsequently, samples were rinsed three times with sterile water. Water from the last rinse was plated on Luria–Bertani’s (LB) agar to test whether the root surface was successfully disinfected. Surface-sterilized roots (approximately 0.5 g) were ground by a mortar and pestle in 5 ml of ddH_2_O. Sterile quartz sand was added to the mortar to improve cell wall disruption. Serial dilutions were plated on LB medium containing 800 μM NaAsO_2_ and incubated at 28 °C for 7 days. Single colonies were picked and restreaked several times to obtain pure isolates. Isolates were stored on LB medium at 4 °C.

### Genomic DNA extraction, PCR amplification, and ARDRA

Genomic DNA of the isolates was extracted from isolates grown in 5 mL of LB liquid medium at 28 °C with 150 rpm for 18 h, using the standard phenol-chloroform method described by Chang et al. [21]. The 16S rRNA gene was amplified in a T100 ™ thermal cycler (Bio-Rad, Hemel Hempstead, UK), using a pre-denaturation step at 94 °C for 4 min, followed by 30 cycles of 50 s at 94 °C, 55 s of annealing at 57 °C, 80 s extension at 72 °C, and a final extension for 8 min at 72 °C. The PCR mixture (25 μL) contained 50 ng of DNA template, 1.5 mM MgCl_2_, 2.5 U Taq DNA polymerase (Invitrogen, USA), 1× PCR buffer, 100 pmol of the primers BSF 8/20: 5′-AGAGTTTGATCCTGGCTCAG-3′; BSR1541/20: 5′-AAGGAGGTGATCCAGCCGCA-3′ [22], and 200 μM of each dNTP. The PCR products were checked by electrophoresis in a 1% (*w*/*v*) agarose gel with 0.5 μg mL^− 1^ ethidium bromide; subsequently, obtained PCR products (1.5 kb) were purified with a Gel Extraction Kit (SBS Genetech, Shanghai, China).

In the amplified rDNA restriction fragment analysis (ARDRA), purified PCR products (about 5 μl) were digested at 37 °C for 4 h, using the restriction enzymes *Hha*I, *Hae*III, *Msp*I, and *Taq*I. Based on colony morphology and the 16S rRNA gene ARDRA pattern, representative isolates were selected for 16S rRNA gene sequencing in an ABI3730XL automatic sequencer at Sangon Biotech (Shanghai, China).

### Amplification of *arsB* and *ACR3(2)* genes

The *arsB* genes were amplified with the degenerate primers darsB1F (5’-GGTGTGGAACATC- GTCTGGAAYGCNAC-3′) and darsB1R (5’-CAGGCCGTACACCACCAGRTACATNCC-3′) [17]. The *ACR*3 (2) genes were amplified with the primers dacr5F (5’-TGATCTGGGTCATGATCTTCCC- VATGMTGVT-3′) and dacr4R (5’-CGGCCACGGCCAGYTCRAARAARTT-3′) [17]. The PCR products were purified with a Gel Extraction Kit (SBS Genetech, Shanghai, China) and sequenced as described above.

### DNA sequencing and phylogenetic analysis

All amplified products were sequenced using an ABI3730XL automatic sequencer at Sangon Biotech (Shanghai, China). Similar sequences were searched using BlastN for 16S rRNA gene and BlastX for *arsB*/*Acr3p*. Sequences from the isolates and reference sequences from GenBank were checked manually and edited to the same lengths, using the software package ClustalX 2.0 [23]. Phylogenetic trees were constructed by the neighbor-joining distance method, using the software package MEGA 5.0 [24]. The reliability of inferred trees was tested with 1000 bootstrap replicates.

### Determination of arsenite resistance and indole-3-acetic acid (IAA) and siderophore production

The minimum inhibitory concentrations (MICs), defined as the lowest concentrations of arsenic as NaAsO_2_ that inhibited growth in chemically defined medium (CDM) broth, were tested in triplicate. Stock solutions of NaAsO_2_ were prepared in ddH_2_O and sterilized. Isolates were inoculated in 3 mL of CDM broth supplemented with 6, 8, 10, 15, 20, 25, 30, 36, 42, and 48 mM NaAsO_2_ and incubated at 28 °C for 4 days. Low salt phosphate (LSP) agar plates without NaAsO_2_ were used as controls. The IAA production was measured as described by Sheng et al. [25], while production of siderophores was evaluated using the chrome azurol-S (CAS) analytical method [26, 27].

### Nucleotide sequence accession numbers

The sequences obtained in this study were deposited in the NCBI GenBank database under accession numbers MF185755–MF185785 for 16S rRNA genes, MF185786–MF185798 for *arsB*, and MF185799–MF185803 for *ACR3(2)*.

### Statistical analysis

Analysis of variance and the Student–Newman-Keuls (SNK) test (*P* < 0.05) were used to compare treatment means. All analyses were performed using SPSS 13.0 for Windows (SPSS Inc., Chicago, USA).

## Results

### Soil physicochemical parameters and metal contents

All soils in the sampling sites were alkaline. However, the pH in the mine area was significantly higher than that in the non-contaminated control site S1 (Table 1). Soil WC in the control site was higher than in the mining area. The highest TN and SOC levels were detected at S1 and the lowest at S5. The highest concentrations of Cu, Cd, Zn, Pb, and As were detected at S3 (Table 1).

**Table 1:** The physico-chemical properties and heavy metal contents of the soils

Sampling site	pH	WC (%)	TN (g kg^− 1^)	SOC (%)	Cu (mg kg^−1^)	Cd (mg kg^− 1^)	Zn (mg kg^− 1^)	Pb (mg kg^− 1^)	As (mg kg^− 1^)
S1	7.55 ± 0.07c	6.26 ± 0.43a	0.25 ± 0.03a	4.76 ± 0.26a	0.12 ± 0.01c	0.07 ± 0.01e	53.4 ± 3.35d	15.6 ± 1.12e	5.36 ± 0.87c
S2	7.78 ± 0.02a	2.32 ± 0.62c	0.12 ± 0.02b	4.37 ± 0.08a	0.88 ± 0.05b	5.15 ± 0.52b	245.7 ± 2.97c	411.3 ± 10.3b	21.3 ± 1.23a
S3	7.71 ± 0.04b	2.88 ± 0.28c	0.10 ± 0.02b	4.23 ± 0.55a	3.40 ± 0.35a	7.99 ± 0.08a	790.4 ± 7.22a	882.5 ± 78.5a	24.5 ± 1.13a
S4	7.63 ± 0.03ab	4.73 ± 0.47b	0.11 ± 0.01b	4.10 ± 0.51a	0.53 ± 0.15b	1.12 ± 0.57d	367.9 ± 6.52b	258.2 ± 33.0c	10.8 ± 2.01b
S5	7.78 ± 0.02a	4.15 ± 0.55b	0.09 ± 0.01b	3.65 ± 0.06a	0.72 ± 0.16b	2.34 ± 0.18c	333.2 ± 45.3b	177.6 ± 15.3d	12.3 ± 0.85b

Data are mean ± SE (*n* = 3); different letters in the same column indicate statistically significant differences (SNK test, *P* < 0.05). *WC* soil gravimetric water content; *TN* total nitrogen, *SOC* soil organic carbon

### Metal concentrations in *P. vittata* roots

Concentrations of Cu, Cd, Zn, Pb, and As in the *P. vittata* roots varied significantly (*P* < 0.05; Table 2). Concentrations of Cu, Cd, Pb, and As were highest at S2, while the Zn level was highest at S3. Compared with the control site, the Cu, Cd, Zn, Pb, and As concentrations were 2.7 to 6.2 (S2), 2.44 to 6.59 (S3), 0.77 to 6.27 (S4), and 2.04 to 6.34 (S5) times higher, respectively.

**Table 2:** Heavy metals concentration in the *P. vittata* roots from different sampling sites

Sampling site	Cu (mg kg^−1^)	Cd (mg kg^−1^)	Zn (mg kg^−1^)	Pb (mg kg^− 1^)	As (mg kg^− 1^)
S1	11.1 ± 2.03c	8.02 ± 1.03c	157.1 ± 35.2b	93.3 ± 6.03d	31.3 ± 10.2c
S2	65.1 ± 5.12a	37.1 ± 3.12a	974.7 ± 65.5a	587.1 ± 22.1a	308.7 ± 12.3a
S3	57.7 ± 4.22a	31.7 ± 6.12ab	1035.4 ± 34.3a	499.3 ± 23.1b	241.6 ± 23.5a
S4	33.2 ± 7.01b	19.5 ± 5.05bc	984.6 ± 83.1a	162.8 ± 36.2d	102.2 ± 10.4b
S5	42.4 ± 4.21b	24.4 ± 3.22ab	995.1 ± 62.6a	344.6 ± 24.5c	125.2 ± 30.7b

Data are mean ± SE (*n* = 3); different letters in the same column indicate statistically significant differences (SNK test, *P* < 0.05)

### Diversity and phylogeny of endophytic bacteria

**Fig. 1:**
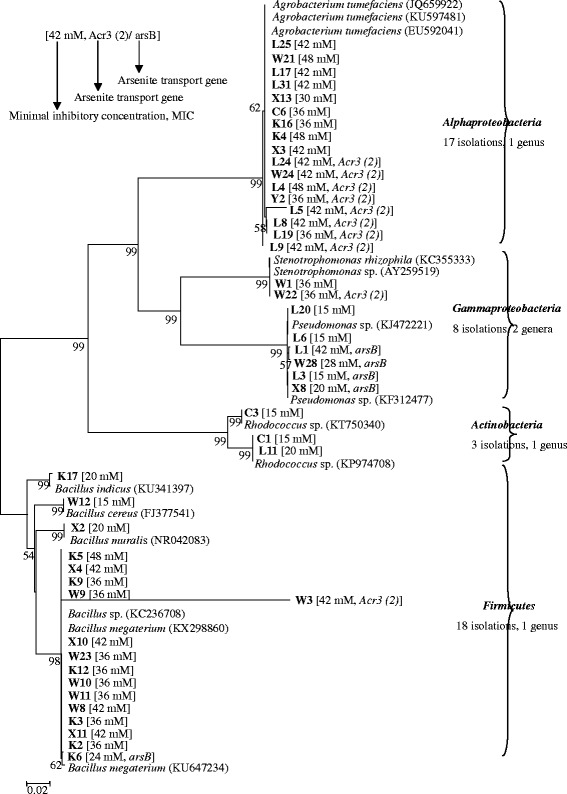
Relationships between the representative isolates and the reference strains based on 16S rRNA gene (~ 1500 bp) phylogenetic analysis. Minimum inhibitory concentration (MIC) for arsenite and the presence of arsenite-resistance genes *ArsB*/ *Acr3(2)* are indicated in squared brackets. Sequences from this study are in bold type. Bootstrap values over 50% are shown on the branching points. The scale bar indicates 2% nucleotide sequence substitution. Isolates with capital L were isolated from the *P. vittata* roots collected from site S5, with X from site S4, with W from site S3, with K from site S2, and with C from site S1

A total of 116 As-resistant endophytic isolates were obtained. To estimate the diversity, the 16S rRNA genes of the isolates were analyzed by ARDRA, resulting in 16 different ARDRA patterns (Table 3). The 46 representative isolates, selected based on colony morphology and ARDRA, carried 16S rRNA genes 99–100% similar with the reference sequences in GenBank. The representative isolates belonged to five genera in four families: *Alphaproteobacteria* (17 isolates, one genus), *Gammaproteobacteria* (eight isolates, two genera), *Actinobacteria* (three isolates, one genus), and *Firmicutes* (18 isolates, one genus) (Fig. 1). Fifteen of the isolates were obtained from the slag heap site (S5), 12 from the tailing dam site (S3), seven from the ore charge heap site (S4), nine from the ore outlet site (S2), and three from the control site (S1) (Fig. 1). Most of the isolates from the slag heap site belonged to the genus *Agrobacterium*. The majority of the isolates from the tailing dam site, the ore charge heap site, and the ore outlet site belonged to the genus *Bacillus*, while the isolates from the control site belonged to the genera *Rhodococcus* and *Agrobacterium*.

**Table 3:** Plant growth promoting characteristics of endophytic bacterial isolates from *P. vittata*. L.

Isolate (closest relative sequence)	% identity	ARDRA type	IAA (μg ml^−1^)^a^	Siderophore^b^
L9 (*Agrobacterium tumefaciens*)	99	I	46.6 ± 4.36	–
L17 (*Agrobacterium tumefaciens*)	99	I	95.0 ± 4.88	–
L25 (*Agrobacterium tumefaciens*)	99	I	42.5 ± 9.42	+
L31 (*Agrobacterium tumefaciens*)	99	I	92.3 ± 8.66	+
W21 (*Agrobacterium tumefaciens*)	99	I	32.8 ± 2.58	+
X13 (*Agrobacterium rhizogenes*)	99	II	–	++
C6 (*Agrobacterium rhizogenes*)	99	II	81.2 ± 4.77	++
Y2 (*Agrobacterium rhizogenes*)	99	II	41.4 ± 3.33	–
K4 (*Agrobacterium* sp.)	99	III	44.4 ± 6.52	+++
K16 (*Agrobacterium* sp.)	99	III	44.2 ± 5.51	+
X3 (*Agrobacterium* sp.)	99	III	44.5 ± 7.88	+
L24 (*Agrobacterium* sp.)	99	III	46.0 ± 2.22	–
W24 (*Agrobacterium* sp.)	99	III	–	–
L19 (*Agrobacterium* sp.)	99	III	47.6 ± 5.54	+
L4 (*Rhizobium* sp.)	99	IV	34.8 ± 3.11	+
L5 (*Rhizobium* sp.)	99	IV	60.9 ± 9.66	+++
L8 (*Rhizobium* sp.)	99	IV	103.9 ± 11.2	++++
W1 (*Stenotrophomonas rhizophila*)	99	V	47.8 ± 4.75	++
W22 (*Stenotrophomonas* sp.)	99	VI	20.4 ± 3.36	–
L20 (*Pseudomonas* sp.)	100	VII	34.4 ± 2.28	–
X8 (*Pseudomonas* sp.)	100	VII	120.3 ± 9.61	+
L6 (*Pseudomonas oryzihabitan*s)	99	VIII	45.9 ± 3.25	+
L1 (*Pseudomonas oryzihabitan*s)	99	VIII	32.4 ± 4.44	++
W28 (*Pseudomonas putida*)	99	IX	38.0 ± 4.25	++
L3 (*Pseudomonas putida*)	99	IX	55.1 ± 4.01	++
C1 (*Rhodococcus* sp.)	99	X	29.5 ± 3.15	++
C3 (*Rhodococcus* sp.)	99	X	65.6 ± 5.55	–
L11 (*Rhodococcus equi*)	99	XI	98.8 ± 8.16	+++
K17 (*Bacillus indicus*)	99	XII	–	+
W12 (*Bacillus cereus*)	99	XIII	49.2 ± 2.17	+
X2 (*Bacillus murali*s)	99	XIV	22.9 ± 5.43	–
K5 (*Bacillus subtilis*)	99	XV	31.7 ± 4.22	++++
W9 (*Bacillus megaterium*)	99	XV	34.6 ± 1.78	++
W23 (*Bacillus megaterium*)	99	XV	–	+
X10 (*Bacillus megaterium*)	99	XV	99.6 ± 12.7	+++
K2 (*Bacillus* sp.)	99	XVI	53.1 ± 4.33	++
K3 (*Bacillus* sp.)	99	XVI	43.6 ± 3.16	–
K12 (*Bacillus* sp.)	100	XVI	–	–
K6 (*Bacillus* sp.)	100	XVI	81.4 ± 2.44	++
W8 (*Bacillus* sp.)	99	XVI	56.0 ± 2.08	+++
W10 (*Bacillus* sp.)	99	XVI	54.6 ± 7.03	+++
W11 (*Bacillus* sp.)	99	XVI	–	–
X4 (*Bacillus* sp.)	99	XVI	39.0 ± 4.21	++++
X11 (*Bacillus* sp.)	99	XVI	17.7 ± 3.22	–
K9 (*Bacillus* sp.)	99	XVI	14.9 ± 0.31	++
W3 (*Bacillus* sp.)	99	XVI	15.2 ± 0.90	+

^a^IAA production: - = not detectable; ^b^ Siderophore production: +, little; ++, low; +++, moderate; ++++, high

### Arsenite resistance levels and plant growth-promoting properties of the isolates

In the minimum inhibitory concentration (MIC) test for arsenite, all strains grew in 15 mM, 36 in 36 mM, 19 in 42 mM, and 5 in 48 mM NaAsO_2_ (Fig. 1). All 17 *Agrobacterium tumefaciens* isolates showed high MICs (≥ 30 mM), while the *Rhodococcus* sp. isolates showed low MICs (≥ 15 mM). Among the *Pseudomonas* isolates, the isolates containing *arsB* showed higher MICs than those without *arsB*. The average MIC of the 10 *Acr3(2)*-carrying isolates (40.9 ± 4.5 mM) was higher than that of the five *arsB-*carrying isolates (25.8 ± 2.3 mM). Five of the 19 highly arsenite-resistant isolates (MIC > 42 mM) were obtained from *P. vittata* roots with high arsenic levels (Fig. 1, Table 2). Isolates from the control site showed lower average MIC than the other isolates (Fig. 1).

In total, 28 out of the 46 isolates produced both IAA and siderophores (Table 3). The majority of the isolates produced 17.7–103.9 μg ml^− 1^ IAA. Isolates L8 (*Rhizobium* sp.), L11 (*Rhodococcus equi*), L31 (*Agrobacterium tumefaciens*), and X10 (*Bacillus megaterium*) produced over 90 μg ml^− 1^ of IAA. Two-thirds of the sequenced endophytes produced siderophores. Isolates L8 (*Rhizobium* sp.), K5 (*Bacillus subtilis*), and X4 (*Bacillus* sp.) produced more siderophores than the other isolates.

### Horizontal transfer of the arsenite transporter genes *arsB* and *Acr3(2)*

**Fig. 2:**
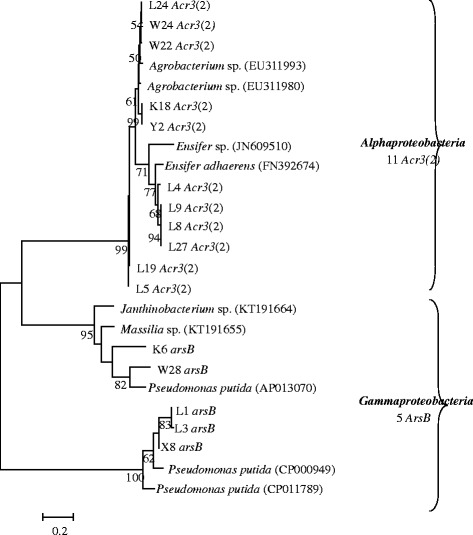
Relationships between the representative isolates and the reference strains based on arsenite transporters *ArsB* and *Acr3(2)*

**Fig. 3:**
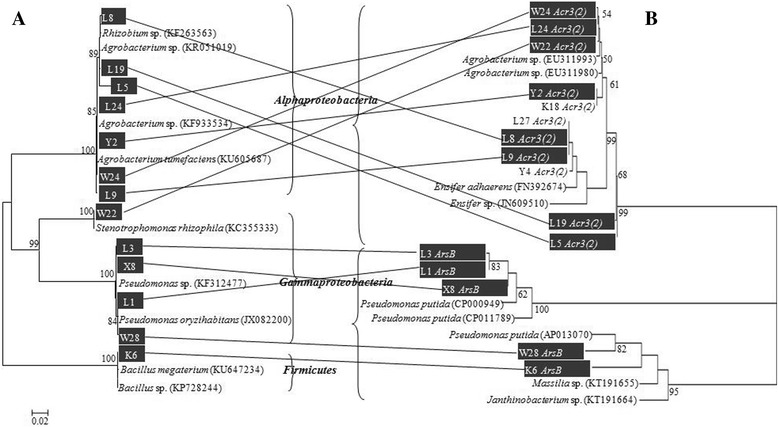
Comparison of 16S rRNA gene (A) and *arsB*/*ACR3(2)* (B) phylogenies. The sequences are subsets from Figs. 1 and 2

A total of 16 arsenite transporter genes, including five *arsB* genes and 11 *ACR3(2)* genes, were successfully amplified (Fig. 2). In the sequence analyses with the BlastX algorithm, the *arsB* and *Acr3(2)* formed separate clusters (Fig. 2). The *arsB* and *Acr3(2)* sequences of the isolates were similar to those from *Gammaproteobacteria* and *Alphaproteobacteria*, respectively. The *Acr3(2)* sequences were divided into separate subgroups, similar to either *Acr3(2)* from *Agrobacterium* sp. or from *Ensifer* sp. *Agrobacterium* sp. L5, L8, L9, and L19 carried *Acr3(2)* similar to those of *Ensifer* sp. (Fig. 3). Gammaproteobacterial *Stenotrophomonas* sp. W22 carried *Alphaproteobacteria* type *Acr3(2)* (Fig. 3). The *Bacillus* sp. K6 carried *arsB* similar to those of the *Gammaproteobacteria* clade (Fig. 3). All of these, possibly horizontally transferred *ACR3*(*2*) and *arsB* genes, were obtained from isolates from the highly As-contaminated mining area.

## Discussion

Plants that accumulate As can provide a specific environment for bacterial endophytes to adapt to high As concentrations [28]. In this work, we isolated and characterized bacterial endophytes from the roots of *P. vittata*, an arsenic hyperaccumulator growing in the Pb-Zn mine tailings in Southwest China [18].

In our study, we isolated 116 As-resistant bacterial endophytes. Despite the small number of isolates, the As-resistant bacterial endophyte community was diverse. The representative isolates were affiliated with *Pseudomonas*, *Stenotrophomonas*, *Rhodococcus*, *Agrobacterium*, and *Bacillus*. Of these, *Agrobacterium*, *Bacillus*, and *Pseudomonas* species have previously been reported as metal-resistant endophytes in other As-contaminated sites [29–31]. Arsenite-resistant *Stenotrophomonas* strains have been isolated from As-contaminated soil and water [32], but, to our knowledge, endophytic arsenite-resistant *Stenotrophomonas* and *Rhodococcus* have not been isolated earlier. Most of the representative isolates belonged to *Agrobacterium* (phylum *Proteobacteria*) and *Bacillus* (phylum *Firmicutes*). Similarly, Ni-resistant bacterial endophytes from *Alyssum bertolonii* belonged mainly to *Firmicutes* and *Actinobacteria* [33]. Moreover, 14 endophytes isolated from *Sedum alfredii* belonged mainly to *Proteobacteria* (57%) and *Firmicutes* (43%) [34]. Taken together, these results suggest that the predominant bacterial endophytes in heavy metal-accumulating plants belong to *Firmicutes*, *Proteobacteria*, and *Actinobacteria* [35]. These highly adapted groups may accelerate the degradation of complex soil compounds by producing extracellular enzymes, and thus stimulate the growth of other bacteria in As-contaminated rhizosphere soil [36].

Endophytic bacteria can increase the growth and development of plants growing in contaminated sites and their metal resistance by producing the phytohormone IAA and siderophores [37–39]. Siderophores are organic molecules with a high affinity for Fe(III) ions and form complexes with other metals, thus participating in nutrient mobilization and metal availability to plants [40, 41]. Bacterial siderophore production may be stimulated by heavy metals and can alleviate the toxicity of heavy-metals to plants by increasing the supply of iron [41]. Arsenic-resistant strains isolated from *P. vittata* have been reported to produce siderophores [39]. Most of the isolates in our study produced IAA or siderophores, indicating that the endophytes may help their host plant to colonize As-contaminated sites [38].

In our study, *P. vittata* roots from sites S2 and S3 (with high As-concentrations) hosted a higher diversity of arsenite-resistant bacteria. In addition, the resistance levels of the isolates were also higher than those among isolates from sites with intermediate and low As concentrations. The 19 strains with arsenite MICs over 40 mM were all isolated from *P. vittata* growing in the Pb-Zn mining area. Previous studies have proposed that high As-contamination levels are likely to exert a strong selective pressure, thereby decreasing microbial diversity [17, 42]. However, in our study, *P. vittata* was widely distributed in the Pb-Zn mine area [18], which may result in the evolution of more bacterial species that are well adapted to highly arsenic-contaminated *P. vittata*. Moreover, Huang et al. [43] reported that in a long-term field site (1951-present), arsenite-resistant endophytic microbial communities of *P. vittata* had had sufficient time to adapt to metal and/or metalloid stress. Zhu et al. [35] found that the diversity of As-resistant endophytes in soil with high arsenic levels was higher than that in less contaminated soil. These results suggest that endophytic bacteria have adapted to high arsenic stress levels and maintained their diversity in *P. vittata* after long-term exposure to high As-levels [44].

Exploring the relations among the degree of arsenite resistance, the distribution and diversity of the arsenite transporter gene families of the bacterial endophytes is a key goal of microbial ecology. The *ACR3(2)* genotypes were previously reported to be predominant over *arsB* in bacterial strains isolated from As contaminated soil [17], which was similar to findings in our study. To cope with arsenic toxicity, multiple sets of arsenic resistance genes and operons were developed in the genome of nearly every bacterial species sequenced to date. For example, the full genome sequence of strain *Brevibacterium linens* AE038–8 contained three *ars* operons (*arsC*, *ACR3* and *arsR*) and two copies of the *arsO* gene [45], *Thiomonas* sp. possessed two operons (*aio* and *ars* system) [46], while *Rhodopseudomonas palustris CGA009* carried three sets of arsenic resistance determinants (*ars1*, *ars2*, and *ars3*) on the chromosome [47]. Previous study has reported that soil bacteria could acquire multiple resistance determinants via chromosomal duplication or horizontal gene transfer, allowing them to cope with long-term arsenic toxicity [44]. Surprisingly, in our study, none of the isolates carried both types of arsenite transporter genes, even though both these genes are commonly found on the same operon. The *ACR3* may have a higher affinity to arsenite and a higher rate of arsenite transport than *arsB,* which makes it more effective [44]. Accordingly, compared to the *arsB* gene-containing strains, strains having *ACR3*(*2*) mostly showed higher arsenite resistance in our study.

Horizontal gene transfer (HGT) plays an important role in allowing a microbial community to rapidly adapt to a new environmental stress like heavy metal contamination, and thus could play an important role in the adaptation of the endogenous endophytic community [39]. In our present study, *ACR3(2)* appeared to be more easily transferred than *arsB*, and the transfer would be possibly stimulated by the exponential growth of environmental pollution as proposed in a previous study [48]. Cai et al. [44] have reported that arsenite transporter genes were transferred between *Aeromonas*, *Stenotrophomonas*, and *Comamonas* in highly As-contaminated soils. Similarly, the phylogenetic discrepancies between 16S rRNA genes and *ACR3*(*2*)/*arsB* indicate that *ACR3*(*2*) might have been horizontally transferred, especially in the isolates from *P. vittata* roots containing high As-levels. The HGT process may have occurred under the high arsenic pressure and resulted in increased functional and species diversity [49, 50], which may have practical applications in equipping the natural endophyte populations capable of resisting As and does not require long-term establishment of the inoculant strain.

## Conclusions

We investigated the distribution and diversity of arsenite-resistant endophytic bacteria in *P. vittata* roots, collected from five soils with different levels of As-contamination, and studied the arsenite resistance and arsenic transport genes of the isolates. *Proteobacteria*, *Actinobacteria*, and *Firmicutes* were the predominant taxa. The distribution and diversity of cultivable endophytes were affected by the arsenic concentration in *P. vittata*. A high number of the isolates were resistant to high concentrations of As and multiple heavy metals and showed plant growth-promoting characteristics. Horizontal gene transfer of *ACR3(2)* and *arsB* was detected in some of the isolates from *P. vittata* roots with high As concentrations. Overall, this study provides valuable information about endophytic bacterial species in relation with As transport and As-resistance genes. Our results contribute to the knowledge on the diversity and distribution of As-resistant endophytic bacteria that may be applied in the phytoremediation of As-contaminated sites.

## Abbreviations

ARDRA: Amplified rDNA restriction fragment analysis
As: Arsenic
CAS: Chrome azurol-S
Cd: Cadmium
CDM: Chemically defined medium
Cu: Copper
DNA: Deoxyribonucleic acid
IAA: Indole-3-acetic acid
LB: Luria–Bertani
MICs: Minimum inhibitory concentrations
NCBI: National center for biotechnology information
Pb: Lead
PCR: Polymerase chain reaction
pH: Post hatch
RNA: Ribosomal ribonucleic acid
SLP: Salt low phosphate
SOC: Soil organic carbon
TN: total nitrogen
WC: Water content
Zn: Zinc
